# Teaching Foundation Doctors How to Manage Tracheostomy Emergencies

**DOI:** 10.7759/cureus.94846

**Published:** 2025-10-18

**Authors:** Ashley Wragg, Joe Gleeson-Buddhdev, Thomas Sharp, Reshad Khodabocus

**Affiliations:** 1 Medical Education and Simulation, The Mid Yorkshire Teaching NHS Trust, Wakefield, GBR; 2 Emergency Medicine, The Mid Yorkshire Teaching NHS Trust, Wakefield, GBR

**Keywords:** foundation doctors, medical education, near peer teaching, simulation, tracheostomy, tracheostomy complications

## Abstract

Background and objective

Tracheostomies are increasingly common in both acute and long-term clinical settings. As more and more patients with tracheostomies are being managed on general wards, Foundation Year 1 (FY1) doctors - often the first responders - require competency in managing life-threatening tracheostomy emergencies. This study aimed to investigate whether a short, targeted educational intervention can improve FY1 doctors' knowledge and confidence in this area.

Methods

A single-centre pilot educational intervention was delivered to 34 FY1 doctors in a UK teaching hospital. The intervention included a 30-minute lecture on surgical airway anatomy and the National Tracheostomy Safety Project (NTSP) algorithm, followed by small-group, high-fidelity simulation scenarios. To evidence improvement in knowledge, a quasi-experimental, single-arm study was undertaken, involving assessment of FY1s' knowledge via a written examination at three time points: pre-educational intervention, immediate post-educational intervention, and one month after the educational intervention.

Results

Baseline knowledge was poor, with only eight (25%) of 32 FY1 doctors correctly identifying the difference between tracheostomy and laryngectomy, 14 (41%) correctly identifying the breathing route in laryngectomy patients, and only 11 (10%) of the 103 proposed management actions when managing a tracheostomy emergency actions they suggested were specifically correct. Post-intervention, correct responses rose across all domains, with sustained improvements seen one month later for the answers to the second and third questions.

Conclusions

An educational intervention combining didactic teaching with simulation improved and sustained FY1 doctors’ knowledge and preparedness for dealing with tracheostomy emergencies. Despite limitations such as sample size and knowledge-based assessments, these findings suggest that this was a successful pilot intervention. Future efforts should include broader implementation and performance-based assessments.

## Introduction

Surgical airways are used to enable patients to ventilate via direct access to the lower respiratory tract, and these include tracheostomies and laryngectomies [1]. Tracheostomies provide a definitive airway to allow patients to continue to ventilate primarily via the neck, without the involvement of the upper airway [2]. Short-term tracheostomies are often used as part of a ventilation weaning strategy in intensive care settings. In the long term, they are used as ventilation support for neurological or respiratory diseases [2]. There are up to 20,000 tracheostomies performed annually in the UK [2], and this number is on the rise. This has led to an increased number of tracheostomy patients being managed outside of specialist areas like the ICU within hospitals [2,3].

When tracheostomies are not patent, the patient cannot ventilate and will start to asphyxiate. Therefore, any serious complication with a tracheostomy can be life-threatening [2]. These complications fall into two main categories: blockage (e.g., secondary to mucus, foreign bodies, or blood) and dislodgment (e.g., due to displacement, removal, or false passages) [2]. Around 1% of tracheostomy patients experience serious complications at some point [4], and the rate of complications is increasing [5]. Therefore, all staff who may come into contact with tracheostomy patients must be equipped to handle related emergencies.

In the UK, Foundation Year 1 (FY1) doctors may be among the first in attendance for such emergencies before the arrival of an airway expert such as an anaesthetist or ENT surgeon. However, we were concerned that many foundation doctors lack confidence in assessing and managing tracheostomies. Literature analysing this issue mainly comprises single-centre studies with small sample sizes, but does indicate a lack of confidence in this area [6]. Earlier surveys found that most resident doctors were unable to correctly distinguish between tracheostomy and laryngectomy, and a vast majority felt that their training was insufficient [7]. More recent work in paediatrics suggests this problem persists, with a majority of residents still reporting they felt unprepared to manage tracheostomy emergencies [8]. This is despite the introduction of the emergency tracheostomy management algorithm created by the National Tracheostomy Safety Project (NTSP) [9]. Resident doctors are often not aware of such guidelines, and they do not feel confident in using them to manage patients without further training [6]. We therefore aimed to improve FY1 doctors' confidence and knowledge in dealing with tracheostomy emergencies via teaching.

STR1DE and tracheostomies

The Mid Yorkshire Hospitals NHS Trust delivers a tailored teaching programme for FY1 doctors called STR1DE (Simulation, Teaching, and Reflection for FY1 Development & Education). As part of this initiative, FY1 doctors attend six full-day teaching sessions over the course of the year, taught predominantly by near-peer “FY3” and “FY4” clinical fellows in education and simulation, as well as some clinical fellows who are resident doctors in anaesthetics and emergency medicine. We introduced a pilot teaching session about tracheostomy emergencies into the STR1DE curriculum.

Research questions

Our research questions were as follows: (1) Would the teaching session produce a sustained improvement in FY1 doctors’ baseline knowledge about tracheostomies and surgical airways? (2) Would the teaching session improve FY1 doctors’ ability to recall the steps that should be taken when responding to a tracheostomy emergency?

This article was previously presented as a poster session on July 6-7, 2023, at the Association of Anaesthetists Conference in Leeds, UK.

## Materials and methods

Teaching session design

This project, undertaken in March-April 2023, involved a single-centre educational intervention that aimed to improve FY1 doctors' confidence and knowledge about managing tracheostomy emergencies. The intervention was targeted at FY1 doctors (n=34) in a UK teaching hospital. Sessions (Table 1) were taught to small groups of FY1 doctors (six to eight) and taught by clinical fellows in education and simulation, and anaesthetic trainees. Firstly, a 30-minute didactic lecture was delivered, covering the anatomy, basics of surgical airways, and the NTSP algorithm. This was followed by multiple short simulations of tracheostomy emergencies (Appendix 1, Table 2) using a high-fidelity mannikin. The simulations increased in complexity as the session progressed, requiring FY1s to work logically through the NTSP algorithm to diagnose and manage the issue that had arisen.

**Table 1:** Lesson plan for the educational intervention

Time	Activity	Detail
0-30 mins	Intro	Slides and group discussion – tracheostomy v laryngectomy, indications for each
30-40 mins	Emergency algorithm	Talk through the algorithm, then watch the emergency management video, and answer questions
40-60 mins	Moulage	FY1s in pairs are given a short scenario of an unwell patient with a tracheostomy, expected to 1) call for help and put out a crash call, (2) apply high-flow oxygen to the face and over the trache. If time and ability permit, can progress further down the algorithm (suction catheter, remove inner tube, etc). 5-10 mins per pair, depending on group size
60-65 mins	Close	Questions, summary, distribute handouts

**Table 2:** Example facilitator notes for a simulation

Section/action	Notes
Issue	Dislodged tracheostomy (the opening is occluded against the tracheal wall)
Setup	Tracheostomy tube in place (looking displaced), will need a suction catheter and a 10 ml syringe to deflate cuff
Call for help	Should be completed (2222)
Look, listen, and feel	Patient is shifting small amounts of air only
Apply oxygen via face and tracheostomy	Should be completed
Remove the speaking valve	Not present
Remove the inner tube	This does not solve the issue
Pass a suction catheter	Unable - it is hitting the inner tracheal wall
Deflate the cuff	Should be done, makes little difference
Remove the tracheostomy tube	This helps
Discussion points	Following the algorithm logically works through various potential problems!

FY1 doctors would take it in turns to participate in tracheostomy emergency simulations, working in pairs to implement the NTSP algorithm. Following the simulation, the educators provided rapid, personalised, and performance-related feedback to the foundation doctors, highlighting good practice and areas for improvement. The feedback focussed predominantly on the application of the NTSP algorithm, though human factors such as teamworking and communication would be commented upon where appropriate.

The learning outcomes for the session were as follows: explain the difference between a tracheostomy and a laryngectomy, to demonstrate correct initial response to a deteriorating patient with a tracheostomy or a laryngectomy, namely calling for help and correctly applying oxygen, and, optionally, to demonstrate further response to a deteriorating patient with a tracheostomy or a laryngectomy, e.g., removing speaking valve and inner tube, suctioning, removing the tracheostomy entirely. 

Rationale for teaching approach

The facilitators were FY3 and FY4 education fellows and junior anaesthetic trainees, and so they were near-peers of the FY1 participants. Near-peer teaching has long been recognised to have numerous benefits, including enabling role-modelling and acquisition of skills [10]. It was felt that using near-peers would emphasise the point that even non-specialist doctors can take the initial steps when dealing with a tracheostomy emergency. Using simulation with immediate feedback is supported by the Kolb cycle [11], allowing participants a concrete experience in a safe environment, before facilitating immediate reflection and abstract conceptualisation, and active experimentation to improve.

Method of assessment

Participants were asked to rate their level of confidence in dealing with a tracheostomy emergency on a 5-point Likert scale (ranging from "not at all confident" to "very confident") immediately before and after the session. They were also asked whether they had any prior experience of dealing with a tracheostomy emergency.

To determine whether the teaching session had a lasting impact, a single-arm, before-and-after quasi-experimental study was designed. All FY1s who attended teaching were eligible to participate. Participation was optional, and informed consent was obtained from those who did participate. FY1 doctors were asked to complete three short assessments. Assessment One was a pre-intervention examination to assess for baseline knowledge and understanding. Assessment Two was a post-intervention examination, taking place directly after the session for immediate comparison to Assessment One. Assessment Three involved a follow-up examination one month after the educational intervention to assess the retention of knowledge and understanding. 

Each assessment was in the form of a short, written examination, consisting of three questions (Appendix 2). The three questions remained the same across all three assessments, allowing direct comparison of FY1s’ knowledge of tracheostomy anatomy, physiology of breathing, and clinical actions to take in tracheostomy emergencies. The assessments were completed in person immediately before or after teaching.

The assessments were marked by the authors. Questions 1 and 3 relied on short free-text responses. For Question 1 ("What is the difference between a tracheostomy and a laryngectomy?"), answers were reviewed in accordance with pre-set criteria (Appendix 3). These questions were double-marked by two authors. Discrepancies in the mark given were rare and resolved by discussion amongst all authors. For Question 3 ("List five management points when dealing with a tracheostomy emergency."), all management points suggested by participants were compiled into a list. The authors then agreed on each suggested management point, whether it was "specifically correct", "generically correct", or "incorrect" (Appendix 4). Question 2 was multiple choice with an objectively correct answer.

## Results

**Figure 1:**
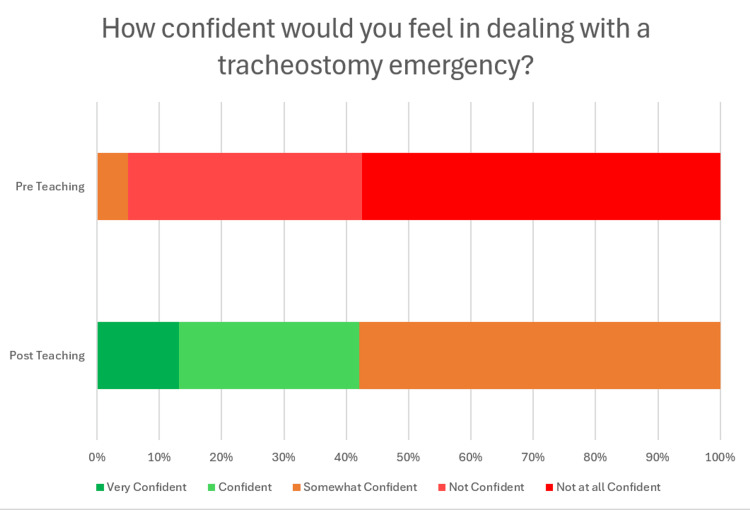
Stacked bar chart showing participants' self-reported confidence in dealing with tracheostomy emergencies before and after the teaching session

The teaching session was very well-received by FY1s - on a 5-point Likert scale (ranging from "very poor" to "excellent"), 20 (61.5%) rated the session as excellent and 12 (38.5%) rated the session as very good. Participants' self-reported confidence improved. Before the session, 23 (57.5%) stated they were not at all confident, 11 (27.5%) were not confident, and two (5%) were somewhat confident. Following the session, 22 (58%) were somewhat confident, 11 (29%) were confident, and five (13%) were very confident (Figure 1).

Only one participant had ever dealt with a tracheostomy emergency in clinical practice and stated that at the time they had felt "not confident" in dealing with it.

The impact of the educational intervention was further assessed by comparing FY1 doctors’ knowledge performance pre-intervention assessment (Assessment One), post-intervention assessment (Assessment Two), and follow-up assessment one month after the educational intervention (Assessment Three). The survey can be viewed in Appendix 2. Assessments One and Two were both completed by 32 FY1s, and 33 FY1s completed Assessment Three.

Question one - ‘What is the difference between a tracheostomy and a laryngectomy?’

This was a free-text question that aimed to assess FY1 doctors’ understanding of different types of surgical airways and the anatomy involved. Answers were categorised as incorrect, correct but inadequate, or fully correct. Examples of an incorrect answer would include “no idea” or “one is in the trachea, one is in the larynx”. Examples of a correct but inadequate answer would include “laryngectomy is permanent” or “no larynx”. An example of a fully correct answer was - “laryngectomy is the removal of the larynx, and a tracheostomy is a stoma inserted into the trachea”.

**Figure 2:**
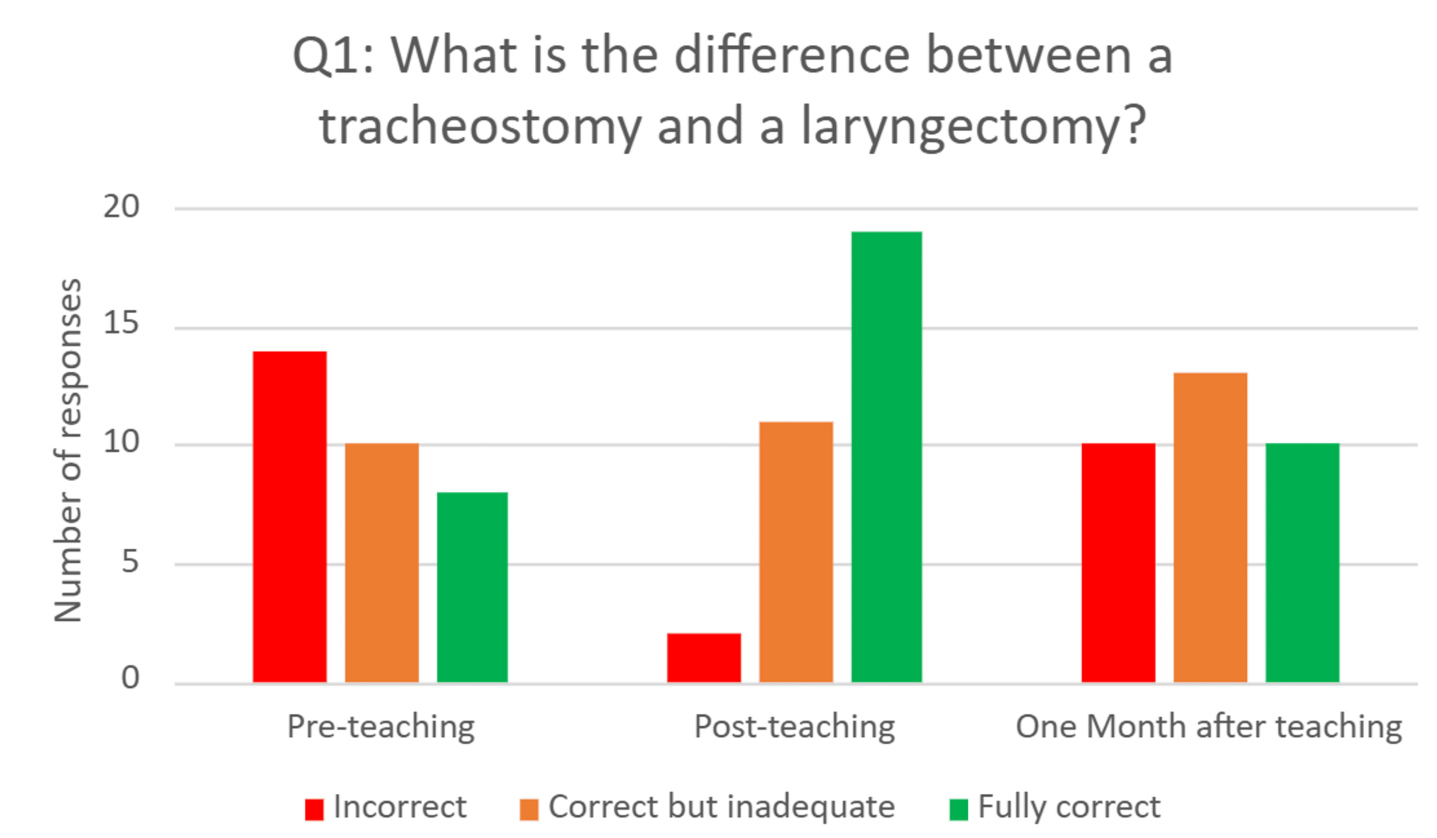
Bar chart showing responses to Q1 before, immediately after, and one month after the educational intervention

Pre-intervention, only eight (25%) FY1 doctors answered the question fully correctly, 10 (31.2%) were correct but inadequate, and 14 (43.7%) FY1s answered incorrectly. Immediately after the intervention, 19 (60%) were fully correct, 11 (34%) were correct but inadequate, and only two (6%) were incorrect. On reassessment one month after the intervention, 10 (30%) were fully correct, 13 (40%) were correct but inadequate, and 10 (30%) answered incorrectly (Figure 2).

​​​​Question two- ‘Can patients with laryngectomies breathe through their mouths, their necks, or both?’ 

This question required participants to select one answer from the answers provided: 1) their mouths, 2) their necks, or 3) both. This question aimed to assess FY1 doctors’ understanding of the anatomy of a laryngectomy and how this affects the physiology of breathing. This is important knowledge as it affects where oxygen therapy should be applied in an emergency. The correct answer was ‘their neck’.

**Figure 3:**
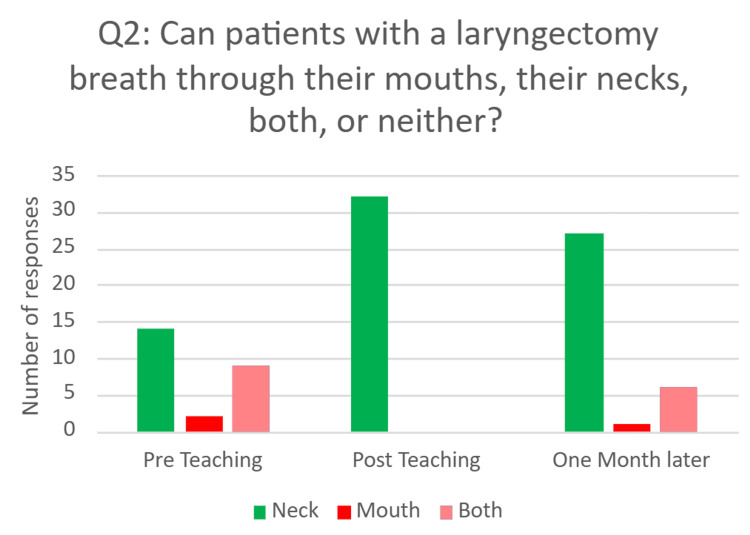
Bar chart showing responses to Q2 before, immediately after, and one month after the educational intervention

Pre-intervention, 14 (56%) answered correctly. This increased to 32 (100%) immediately after teaching, and 27 (78%) answered correctly one month post-intervention (Figure 3).

Question three - ‘List five actions you would take when managing a patient experiencing a tracheostomy emergency’.

This was a free-text question, where FY1 doctors listed five actions they would take when managing a tracheostomy emergency (Figure 3). This question aimed to assess their knowledge of managing a tracheostomy emergency. Answers were categorised as incorrect, generically correct, or specifically correct. An example of an incorrect answer was “Apply oxygen to the neck only”. Examples of generically correct answers included “Call for help” and “Pull the emergency buzzer”. Examples of specifically correct answers included “Follow tracheostomy algorithm” and “Remove the inner tube”.

**Figure 4:**
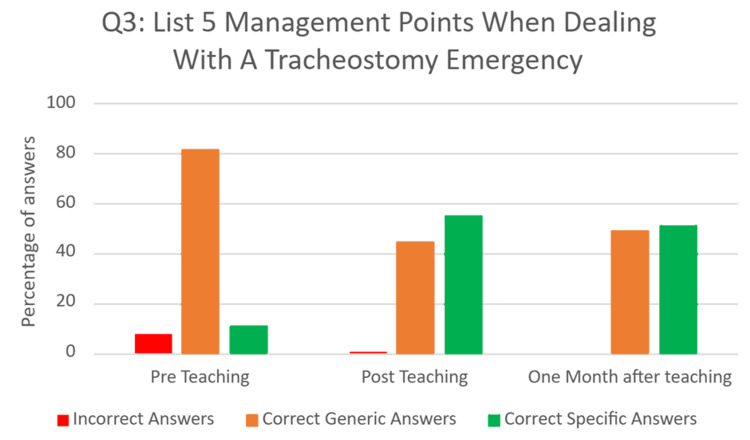
Bar chart showing responses to Q3 before, immediately after, and one month after the educational intervention

Pre-intervention, only 11 (10%) were specifically correct, with 84 (81%) answers generically correct, and eight (7.7%) incorrect answers. Immediately post-intervention, 81 (55%) answers were specifically correct, 65 (44%) were generically correct, and one (0.7%) answer was incorrect. On reassessment one month after the educational intervention, 53 (51%) answers were specifically correct, 51 (49%) were generically correct, and no answers were incorrect. These results demonstrate an increase in specific knowledge and understanding of management of tracheostomy emergencies following the educational intervention (Figure 4).

## Discussion

Previous research has highlighted limited confidence and knowledge among resident doctors in managing tracheostomy emergencies [6-9]. The findings of this study provide additional support for this, with no FY1s rating themselves as "confident" or "very confident" before the educational intervention. Furthermore, our direct assessment of FY1 doctors’ knowledge of the anatomy of surgical airways, physiology of breathing with a surgical airway, and management of tracheostomy emergencies demonstrated a poor baseline. Before the educational intervention, only eight (25%) participants could explain the difference between a tracheostomy and a laryngectomy; almost half did not understand the key fact that patients with laryngectomies can breathe only through the front of their necks, and only 11 (10%) of the actions they suggested were specifically correct for the situation. This adds to the evidence that further training of resident doctors is required to equip them to manage patients with surgical airways.

Considering the small sample size in this pilot study, sustained improvements were observed in responses to Questions Two and Three following the educational intervention. Similar findings have been reported in other tracheostomy education studies, with caregivers showing significant increases in confidence after participating in simulated tracheostomy emergency scenarios [12], and improved knowledge, teamwork, and confidence among nurses and junior doctors following interprofessional workshops [13].

This educational intervention was effective for several reasons. It was developed and facilitated by clinical fellows who possessed expertise in both education and simulation, as well as additional experience in airway management. This combination ensured the programme was grounded in current educational theory while also reinforcing learning with specialist clinical knowledge and personalised feedback. The use of mixed teaching methods further strengthened the intervention: a concise didactic lecture provided a foundation of core knowledge, which was then consolidated through practical application in simulated scenarios. This structure enabled FY1 doctors to first acquire essential principles before applying them to realistic clinical contexts.

High-fidelity, immersive simulation was particularly valuable. Repeated exposure to emergency tracheostomy scenarios and the NTSP algorithm allowed participants to practise in a safe environment, progressively building knowledge and confidence as scenario complexity increased. The next logical step is to expand this intervention beyond FY1 doctors, aiming to support a broader group of clinicians and ultimately enhance outcomes for tracheostomy patients throughout the trust. This aligns with national improvement programmes, which have demonstrated that structured, system-wide initiatives reduce incident severity and enhance tracheostomy safety [3,14]. Looking forward, additional educational approaches may further enhance learning. Simulation has been shown to improve performance in high-pressure situations across various specialties [15], while emerging technologies like virtual reality present scalable opportunities for delivering tracheostomy training [16-18].

It is important to note the significant decline in scores for Question One, which assessed understanding of airway anatomy. Although fully correct responses increased from eight (25%) before the intervention to 19 (60%) immediately afterward, this dropped to 10 (30%) one month later, nearly returning to baseline. In contrast, retention was stronger for Question Two, which evaluated applied anatomical knowledge: correct answers rose from 14 (41%) pre-teaching to 32 (100%) post-teaching, and remained relatively high at 27 (78%) after one month. Question Three, which assessed specific emergency management steps, showed the strongest retention, with specific correct answers increasing from 11 (10%) pre-teaching to 81 (55%) post-teaching, and only slightly declining to 53 (51%) at follow-up. These differences indicate that applied and procedural knowledge was more resistant to decline over time compared to purely conceptual information.

This pattern aligns with existing literature on simulation-based education, which shows that while simulation can lead to significant short-term improvements in knowledge and confidence, these benefits often diminish over time without ongoing reinforcement. For instance, Shrestha et al. [19] found that junior doctors’ advanced cardiac life support skills dropped significantly within 30 days post-training, and Moretti et al. [20] observed basic life support skill deterioration within a similar timeframe. A decline in knowledge over time is therefore to be expected after a single teaching session, and so future iterations of our programme may benefit from incorporating refresher sessions to support longer-term retention.

Limitations

This study has several limitations. Firstly, it was conducted at a single centre with a small sample size, using convenience sampling based solely on attendance at the teaching session. This limits the generalisability of the findings. Additionally, the limited number of participants meant that it was not possible to demonstrate formal statistical significance in the observed improvements. These factors should be considered when interpreting the results of this pilot project.

Secondly, the assessment tool used was brief and unvalidated, which may have limited its ability to fully capture the depth of participants’ understanding or their ability to apply knowledge in complex scenarios. While steps were taken to standardise the marking of the assessments, interpreting free-text responses inevitably introduces subjectivity.

Thirdly, there was no control group, making it impossible to account for other factors that may have improved participants' knowledge. In particular, participants could have had clinical exposure to tracheostomy patients between the teaching session and the follow-up assessment. Furthermore, the Hawthorne Effect [21] could have resulted in participants independently improving their knowledge about tracheostomies, knowing that they would be asked to complete assessments about them.

Finally, our evaluation focused primarily on knowledge retention, corresponding to Level 2 of the Kirkpatrick model of training evaluation [22]. While simulation-based assessment could have allowed us to explore behaviour change (Kirkpatrick Level 3), we opted not to pursue this to preserve time for teaching other essential topics within the STR1DE curriculum. Conducting multiple formal simulations purely for assessment would have reduced the breadth of the educational programme and may not have been well received by FY1 participants, who value the variety and practical relevance of the sessions. Further research is required to assess the impact on patient outcomes (Kirkpatrick Level 4).

## Conclusions

This short educational intervention, which integrated didactic teaching with high-fidelity simulation, enhanced FY1 doctors’ knowledge of tracheostomies and the management of tracheostomy emergencies. Some of these gains were retained one month later. The sustained improvement, especially in applied emergency management steps, highlights the value of simulation-based training in preparing doctors for critical airway scenarios they may encounter on general wards. While there was some decline in conceptual knowledge over time, the overall positive effect suggests that incorporating such focused sessions into foundation teaching could be beneficial. Future initiatives should investigate refresher training and extend the intervention to other trainee groups, while also assessing behavioral changes and patient outcomes to optimise its clinical effectiveness.

## Appendices

Appendix 1 - List of simulated scenarios

**Table 3:** Table showing the issue and solution for each simulation scenario used

Scenario	Issue	Solution
1	Inner tube blocked by secretions	Remove the inner tube
2	Dressing placed over the tracheostomy site	Remove dressing
3	Secretions downstream from tracheostomy	Suction with the suction catheter
4	Dislodged tracheostomy	Remove tracheostomy tube
5	Opioid overdose	Ventilate via tracheostomy, continue ABCDE assessment, and give naloxone

Appendix 2 - Assessment tool

1 - What is the difference between a tracheostomy and a laryngectomy?

2 - Can patients with a laryngectomy breathe through their mouths, their necks, both, or neither?

3 - List five management points when dealing with a tracheostomy emergency.

Appendix 3 - Marking criteria for Question 1

Answers will be marked "fully correct" if they thoroughly explain the anatomical difference between a tracheostomy and a laryngectomy (there must be a reference to both the removal of the larynx in a laryngectomy and the creation of a stoma in the trachea for a tracheostomy).

Answers will be marked "partially correct" if the answer is factually correct but does not thoroughly explain the anatomical difference between a tracheostomy and a laryngectomy.

Answers will be marked "incorrect" if any aspect of the answer is factually incorrect.

Appendix 4 - Actions listed in response to Question 3 and marking category allocated

**Table 4:** Table showing all answers given in response to Question 3, and the marking code allocated to each answer

Answer given	Coding allocated
Apply oxygen to neck and mouth	Specifically correct
Ensure trache still sited	Specifically correct
Change tracheostomy	Specifically correct
Check for obstruction	Specifically correct
Remove inner tube	Specifically correct
Check algorithm	Specifically correct
Remove caps/valves	Specifically correct
Check inner tube for obstructions/secretions/patency	Specifically correct
Deflate cuff	Specifically correct
Call for help/escalate to senior	Generically correct
Apply oxygen (no comment mouth or neck)	Generically correct
Suction (without further clarification)	Generically correct
Sit up	Generically correct
A-E Assessment	Generically correct
CXR	Generically correct
ABG	Generically correct
Check sats	Generically correct
Chest physio	Generically correct
Check if breathing	Generically correct
Check airway patency	Generically correct
Apply oxygen to neck	Incorrect
Apply oxygen to mouth	Incorrect
